# The Activation of Muscarinic Acetylcholine Receptors Protects against Neuroinflammation in a Mouse Model through Attenuating Microglial Inflammation

**DOI:** 10.3390/ijms251910432

**Published:** 2024-09-27

**Authors:** Kaichun Wang, Yuanyuan Xie, Xixiang Chen, Xiaoyan Ouyang, Lanxue Zhao, Hongzhuan Chen, Jianrong Xu

**Affiliations:** 1 School of Integrative Medicine, Shanghai University of Traditional Chinese Medicine, Shanghai 201203, China; 12021174@shutcm.edu.cn (K.W.); 22022630@shutcm.edu.cn (Y.X.); 22021505@shutcm.edu.cn (X.C.); 22023539@shutcm.edu.cn (X.O.); 2 Institute of Interdisciplinary Integrative Medicine Research, Shanghai University of Traditional Chinese Medicine, Shanghai 201203, China; 3 Department of Pharmacology and Chemical Biology, Shanghai Jiao Tong University School of Medicine, Shanghai 200025, China; annzoor@sjtu.edu.cn; 4 Shanghai Frontiers Science Center of TCM Chemical Biology, Shanghai University of Traditional Chinese Medicine, Shanghai 201203, China

**Keywords:** neuroinflammation, muscarinic acetylcholine receptors, microglia, neuron

## Abstract

Neuroinflammation is a critical factor that contributes to neurological impairment and is closely associated with the onset and progression of neurodegenerative diseases. In the central nervous system (CNS), microglia play a pivotal role in the regulation of inflammation through various signaling pathways. Therefore, mitigating microglial inflammation is considered a promising strategy for restraining neuroinflammation. Muscarinic acetylcholine receptors (mAChRs) are widely expressed in the CNS and exhibit clear neuroprotective effects in various disease models. However, whether the activation of mAChRs can harness benefits in neuroinflammation remains largely unexplored. In this study, the anti-inflammatory effects of mAChRs were found in a neuroinflammation mouse model. The expression of various cytokines and chemokines was regulated in the brains and spinal cords after the administration of mAChR agonists. Microglia were the primary target cells through which mAChRs exerted their anti-inflammatory effects. The results showed that the activation of mAChRs decreased the pro-inflammatory phenotypes of microglia, including the expression of inflammatory cytokines, morphological characteristics, and distribution density. Such anti-inflammatory modulation further exerted neuroprotection, which was found to be even more significant by the direct activation of neuronal mAChRs. This study elucidates the dual mechanisms through which mAChRs exert neuroprotective effects in central inflammatory responses, providing evidence for their application in inflammation-related neurological disorders.

## 1. Introduction

Neuroinflammation, an inflammatory response in the central nervous system (CNS), arises from various pathogenic factors, such as infections, toxins, and trauma. It is exacerbated by protein misfolding [1], genetic mutations [2], and dysfunctions of the autoimmune system [3] within the CNS, playing a key role in the pathogenesis of neurological diseases. If these conditions are not promptly addressed, the continuous recruitment of immune cells to affected regions can result in persistent inflammation [4]. Such chronic inflammatory response in the CNS has been identified as a leading cause of neurodegenerative diseases, including Alzheimer’s disease (AD) [5], Parkinson’s disease (PD) [6], and multiple sclerosis (MS) [7].

Among the CNS-resident immune cells, microglia play the primary role in neuroinflammation. These are the myeloid lineage cells tasked with immune surveillance and maintenance of CNS homeostasis [8,9]. In the context of disease, microglia undergo a series of phenotype transitions [10] and rapidly respond to environmental cues [11], through morphological transformation, enhanced phagocytosis, and secretion of pro-inflammatory cytokines [12]. However, hyperactivated microglia lead to deleterious and neurotoxic consequences and dysregulated neuron–glia crosstalk [13]. Functional impairment and sustained activation in microglia contribute to the exacerbation of lesion sites and promotion of neurodegeneration under certain circumstances [14]. Hence, targeting hyperactivated microglia provides promising strategies to attenuate inflammation and mitigate the progression of neurodegeneration.

Cytokines secreted by microglia play a crucial role in regulating the central immune environment. Among them, TNFα and IL-6 are considered to be pro-inflammatory and neurotoxic as a major cause of blood–brain barrier (BBB) damage and neuronal death [15]. Activated microglia also secrete a series of chemokines, such as Ccl2 and Ccl7, which recruit peripheral immune cells like T lymphocytes [16]. In chronic-neuroinflammation-associated BBB impairment, chemokines exacerbate central immune inflammatory infiltration. The elevated expression of cytokines, such as IL-17, promotes the extensive production of encephalitogenic Th17 cells due through inducing differentiation [17]. This pathological process is one of the main pathogenic mechanisms of autoimmune neurological diseases such as MS [18]. Meanwhile, microglia also secrete cytokines with anti-inflammatory effects, such as IL-4 [19] and IL-10 [20], which help restore immune homeostasis when the lesion is resolved, and play a neuroprotective role through tissue repair and regeneration. Compared to the cellular inflammatory response caused by Toll-like receptor activation, the transformation of the anti-inflammatory phenotype of microglia is more complex and has not yet been elucidated. Therefore, finding bioactive substances that can regulate the polarization of microglia and exploring their mechanisms of action is of great significance for neuroinflammation-related neurological diseases.

Muscarinic acetylcholine receptors (mAChRs) belong to the G protein-coupled receptor superfamily and play crucial roles in learning and memory [21]. In the hippocampus and cortex of the brain, the activation of mAChRs modulates synaptic transmission effects on synaptic transmission [22] and circuit activity [23]. Within the mAChR family, M1 muscarinic activation slows neurodegeneration by enhancing adaptive immunity in prion-mediated neuroinflammation [24]. Recent studies have provided unprecedented insights into the physiology, pharmacology, and structure of mAChRs [25,26]. It has been reported that mAChRs are also expressed in central immune cells and play a role in neuroprotection during stroke [27] and brain ischemic injury [28]. However, whether this benefit is associated with an anti-inflammatory effect and the underlying cellular mechanisms remains unknown. Previous research indicates that acetylcholine (ACh), the endogenous agonist of mAChRs, is stored and released in immune cells [29], significantly influencing both innate and acquired immunity [30]. However, endogenous acetylcholine is prone to hydrolysis, which imposes certain limitations on in vitro studies. Pilocarpine is a naturally occurring alkaloid obtained from the leaves of *Pilocarpus jaborandi* or *Pilocarpus microphyllus*, remaining as one of the first-line drugs for ophthalmologists today in the treatment of glaucoma. Our preliminary results indicate that pilocarpine, acting as a partial agonist, exhibits a preference effect in GPCR conformation regulation, selectively recruiting arrestin and activating its downstream signaling pathways [31]. Nevertheless, the specific function of mAChR activation by pilocarpine in microglia and its potential benefits in neuroinflammation require further elucidation.

In this study, we aimed to ascertain the anti-inflammatory consequences of activating mAChRs in an LPS-induced neuroinflammation mouse model. We conducted a thorough investigation into how mAChR activation influences microglial phenotypes and glia–neuron interactions, with the goal of defining the role of mAChRs in modulating immune responses. This research also seeks to establish evidence for using mAChR-targeting drugs in treating neuroinflammation.

## 2. Results

### 2.1. The Activation of Muscarinic Receptors Attenuated Neuroinflammation in an LPS-Induced Mouse Model

**Figure 1:**
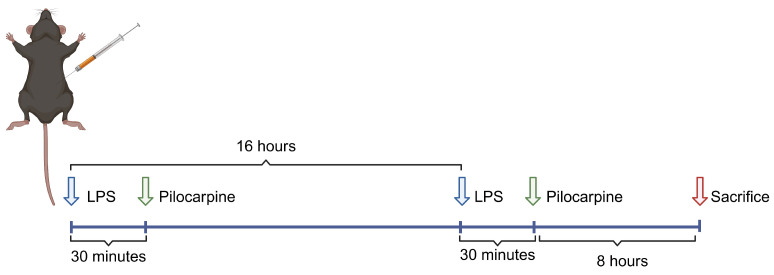
Schematic representation of the experimental design and timeline followed in this study.

A widely used animal model of neuroinflammation [32,33] was introduced to observe the in vivo anti-inflammatory effect of mAChRs. The administration of pilocarpine, the classic agonist of mAChRs, commenced half an hour post LPS stimulation through intraperitoneal injection as shown in Figure 1.

**Figure 2:**
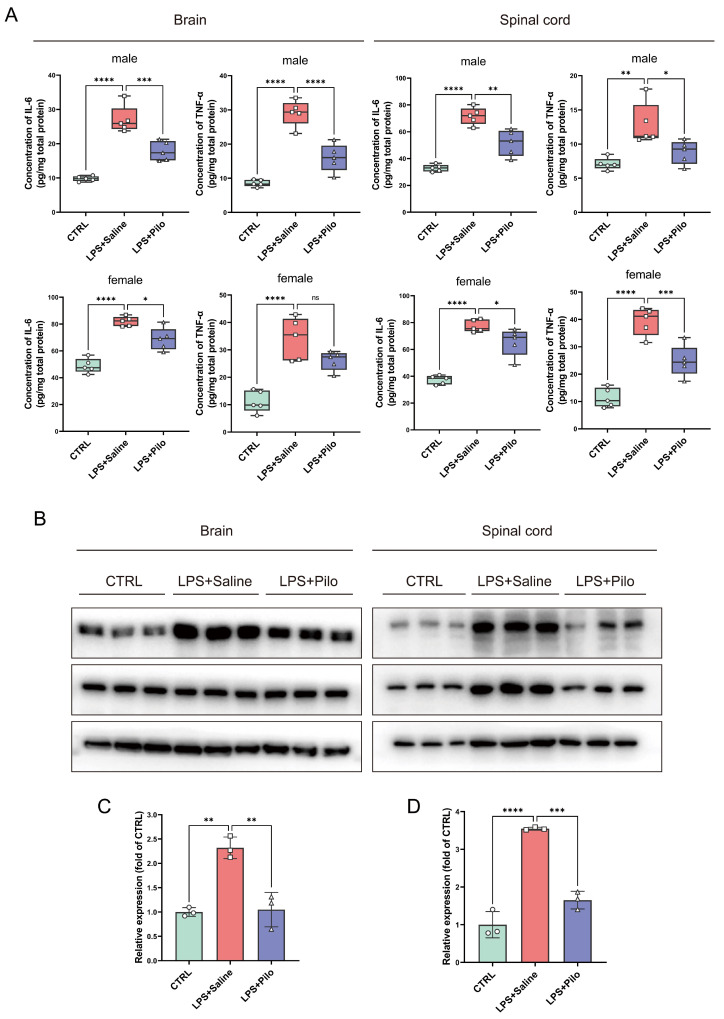
The activation of muscarinic receptors downregulated the expression of pro-inflammatory cytokines and the NF-κB pathway. (**A**) The expression levels of the pro-inflammatory cytokines TNF-α and IL-6 in the brains and spinal cords of the mouse model were examined, respectively, by the ELISA assay (*n* = 5 mice per group). (**B**) The activation of the NF-κB pathway in the CNS of the mouse model were evaluated by Western blot, and the phosphorylation of p65 NF-κB was analyzed and normalized by the control group (**C**,**D**) (*n* = 3 mice per group). * *p* < 0.05, ** *p* < 0.01, *** *p* < 0.001, **** *p* < 0.0001. ns, no significance. Pilo, pilocarpine, see Supplementary Materials File.

To corroborate the variation in inflammatory levels in the central nervous system (CNS), we examined the expression of TNF-α and IL-6 in the brains and spinal cords of the control and LPS-injected mice treated with saline or pilocarpine. The results of the ELISA assay showed that IL-6, the pro-inflammatory cytokines, were significantly elevated from 9.80 ± 0.76 (male) and 48.88 ± 5.41 (female) to 27.03 ± 4.02 (male) and 81.97 ± 3.61 (female) in the brains. The expression in the spinal cords was also increased from 32.85 ± 2.63 (male) and 37.16 ± 2.98 (female) to 71.66 ± 6.47 (male) and 76.28 ± 5.00 (female) in LPS-injected mice. After the administration of pilocarpine, the IL-6 expression decreased to 68.78 ± 8.47 (female) and 17.84 ± 2.86 (male) in the brains, as well as 64.07 ± 9.96 (female) and 51.71 ± 9.66 (male) in the spinal cords (Figure 2A). The expression level of TNF-α also exhibited this trend.

This phenomenon suggested that the activation of mAChRs possessed anti-inflammatory effects in the CNS. Thus, we detected the activity of the NF-κB signaling pathway through analyzing the phosphorylation of p65. As one of the main transcription factors of a series of pro-inflammatory cytokines in the NF-κB family, the phosphorylated p65 is an important sign of the activation and translocation to the nucleus of p65. The immunoblotting results demonstrated that the activation of mAChRs inhibited the phosphorylation of p65 by 45.25% and 46.52% in the brains and spinal cords, respectively, which downregulated the expression of pro-inflammatory cytokines, and subsequently alleviated the CNS inflammatory response (Figure 2B–D).

Together, the decline in the expression of pro-inflammatory cytokines and the activation of the NF-κB pathway proved that the activation of mAChRs attenuated neuroinflammation in vivo.

### 2.2. The Activation of Muscarinic Receptors Regulated the Expression Level of Cytokines in the Brains and Spinal Cords of Mouse Neuroinflammation Models

**Figure 3:**
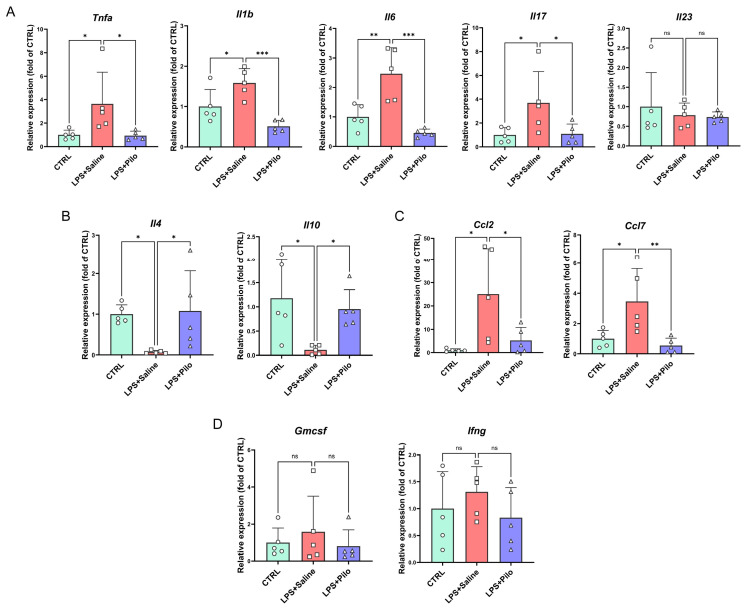
The activation of muscarinic receptors regulated the concentration of a series of cytokines in the brains of the neuroinflammation mouse model. The expression levels of pro-inflammatory cytokines (**A**), anti-inflammatory cytokines (**B**), chemokines (**C**), and M1-polarization-associated cytokines (**D**) were detected by qPCR in the brains of the neuroinflammation mouse model. All data represent mean ± SD from five different experiments. * *p* < 0.05, ** *p* < 0.01, *** *p* < 0.001. ns, no significance. Pilo, pilocarpine.

**Figure 4:**
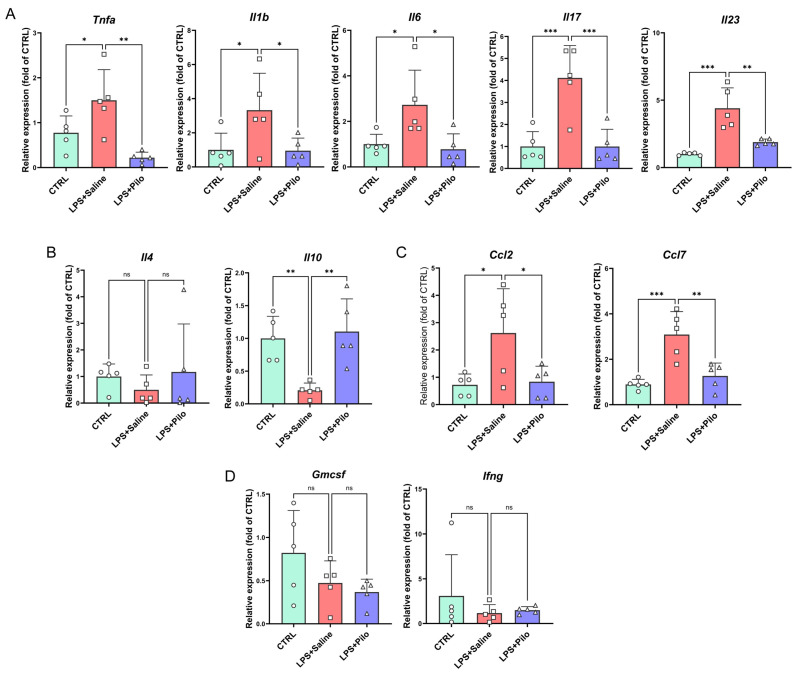
The activation of muscarinic receptors regulated the concentration of a series of cytokines in the spinal cords of the neuroinflammation mouse model. The expression levels of pro-inflammatory cytokines (**A**), anti-inflammatory cytokines (**B**), chemokines (**C**), and M1-polarization-associated cytokines (**D**) were detected by qPCR in the spinal cords of the neuroinflammation mouse model. All data represent mean ± SD from five different experiments. * *p* < 0.05, ** *p* < 0.01, *** *p* < 0.001, ns, no significance. Pilo, pilocarpine.

To explore the atlas of the central immune response, we further examined the expressions of microglia- and non-microglia-derived cytokines with pro- or anti-inflammatory effects. The results of transcriptional level detection indicated that the increased expression levels of a series of pro-inflammatory cytokines including TNF-α, IL-1β, IL-6, and IL-17 were downregulated by the administration of pilocarpine in the brain tissues of the neuroinflammation mouse model (Figure 3A). Additionally, the expression of another pro-inflammatory cytokine, IL-23, was also observed in the spinal cords (Figure 4A). Meanwhile, the anti-inflammatory cytokines IL-4 and IL-10 were detected to define the central immune landscape regulated by the activation of mAChRs. The reduction in IL-10 was reversed by pilocarpine, which suggested the onset of anti-inflammatory action (Figure 3B and Figure 4B).

Moreover, Ccl2 and Ccl7, two of the microglia-secreted chemokines regulating the recruitment of peripheral immune cells to the brain, were remarkably increased in LPS-induced mice and decreased after the activation of muscarinic receptors (Figure 3C and Figure 4C), indicating that the central immune inflammatory infiltration caused by activated microglia is inhibited. Another two lymphocyte-derived immune modulatory cytokines, GM-CSF and IFN-γ, with the function of M1-polarization induction and phenotype transition in microglia, showed no obvious change in expression (Figure 3D and Figure 4D). This result reflected an absence of adaptive immunity in the early stage of neuroinflammation.

Together, these findings suggested that the activation of mAChRs reversed the abnormally elevated expression of microglia-derived cytokines and chemokines and displayed an anti-inflammatory effect in the CNS.

### 2.3. The Levels of Inflammation in Primary Microglia Were Rescued by the Activation of Muscarinic Receptors

**Figure 5:**
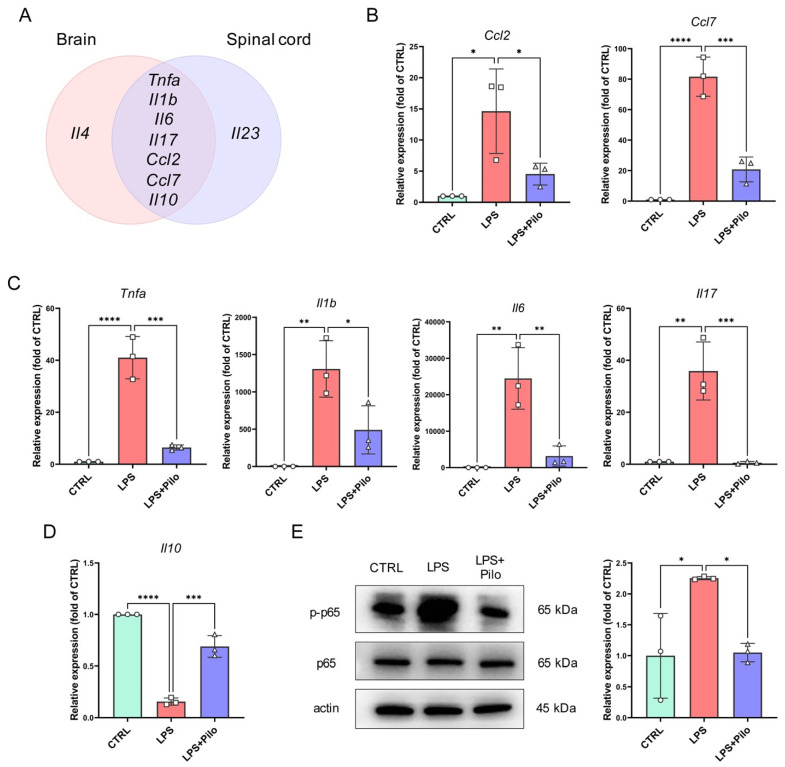
The differentially expressed cytokines were primarily attributed to microglial reaction in neuroinflammation and attenuated by the activation of muscarinic receptors. (**A**) A Venn diagram of differentially expressed genes demonstrating that the seven genes share commonalities in both the brains and spinal cords. (**B**–**D**) The expression levels of these seven genes were detected in the primary microglia by qPCR. (**E**) The activation of the NF-κB pathway in the primary microglia were evaluated by Western blot, and the phosphorylation of p65 NF-κB was analyzed and normalized by the control group. All data represent mean ± SD from three different experiments. * *p* < 0.05, ** *p* < 0.01, *** *p* < 0.001, **** *p* < 0.0001. Pilo, pilocarpine.

A further analysis of the commonalities and differences in cytokines and chemokines in the brains and spinal cords significantly regulated by the activation of mAChRs showed that TNF-α, IL-1β, IL-6, IL-17, Ccl2, Ccl7, and IL-10 were influenced in both the brain and spinal cord, while IL-4 and IL-23 were not (Figure 5A). To investigate the effect of the specific activation of muscarinic receptors in microglia, we detected the expression of the common seven cytokines and chemokines in LPS-stimulated primary microglia after the administration of pilocarpine. The results showed that all these cytokines varied in primary microglia, indicating that the activation on mAChRs exerted anti-inflammatory effects on microglia during neuroinflammation (Figure 5B–D).

In addition, we also examined the phosphorylation of p65, the marker of the activation of the NF-κB pathway. The Western blotting results demonstrated that this inflammation-associated pathway was significantly activated in microglia after stimulation of LPS and was largely alleviated by pilocarpine (Figure 5E).

Together with the above experimental results, the mAChRs distributed on microglia are potential targets in neuroinflammation with effective anti-inflammatory properties.

### 2.4. Pilocarpine-Attenuated Microgliosis in Mouse Neuroinflammation Model

**Figure 6:**
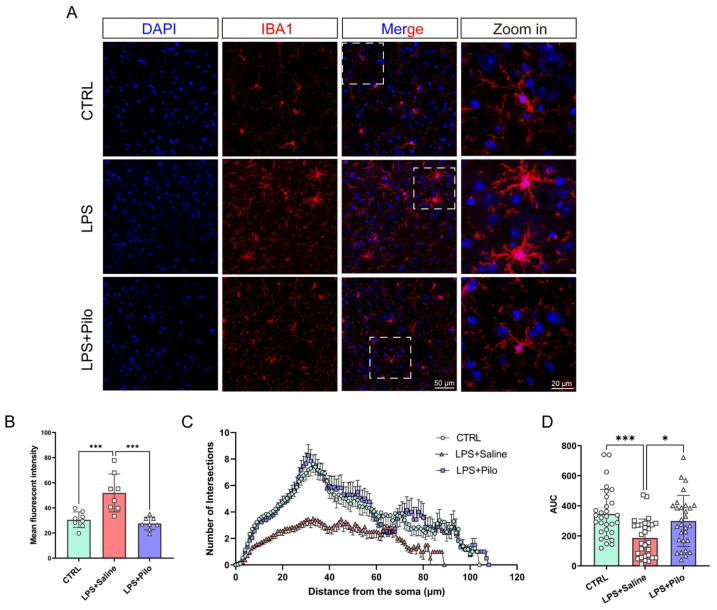
The neuroinflammation-associated microgliosis and microglial activation were attenuated by the activation of muscarinic receptors. (**A**) The brains from the control and LPS-injected mice treated with saline or pilocarpine were stained with DAPI (blue) and anti-Iba1 antibodies (red). Scale bar = 50 μm, 20 μm (zoomed in). (**B**) The distribution of microglia in the brains were detected by the fluorescent intensity ratio of Iba1 and DAPI. (**C**,**D**) The microglial response to inflammatory stimuli was evaluated by the Sholl assay and total area under the curve analyses. All data represent mean ± SD from at least three different experiments. * *p* < 0.05, *** *p* < 0.001, ns, no significance. Pilo, pilocarpine.

Microgliosis [34] is one of the pathological phenotypes of neuroinflammation and reflects the number, distribution density, and soma complexity of microglia following a stress response. An immunofluorescence staining assay demonstrated that the number of microglia was observably increased in the brains of the LPS-induced mouse model with a higher mean fluorescence intensity of IBA1 (Figure 6A,B), which was attenuated by the administration of pilocarpine. Meanwhile, as shown in the zoomed-in list of images, the microglial complexity was also rescued with the finer branches and smaller somas (Figure 6A).

In order to evaluate the changes in microglial state during the inflammatory process with quantitative indicators, we introduced the Sholl assay to analyze the intersections between branches and concentric circles centered on the soma, beginning at 0 μm and increasing 1 μm with every circle. The Sholl curves of each group of mice showed that the maximum number of intersections was several folds smaller than the control and the maximum distance from the soma was reduced, describing the retraction of activated microglia in neuroinflammation (Figure 6C). After the treatment of pilocarpine, the intersections and distance were substantially recovered. An analysis of area under the curve (AUC) showed that the stimulation of LPS led to a reduction from 345.7 ± 29.7 to 186.5 ± 22.9, while the activation of mAChRs significantly elevated the AUC to 298.9 ± 31.7 (Figure 6D), which suggested that microgliosis and morphological alteration of activated microglia can be regulated by mAChRs.

### 2.5. Pilocarpine Improved Morphological Changes in Activated Microglia

**Figure 7:**
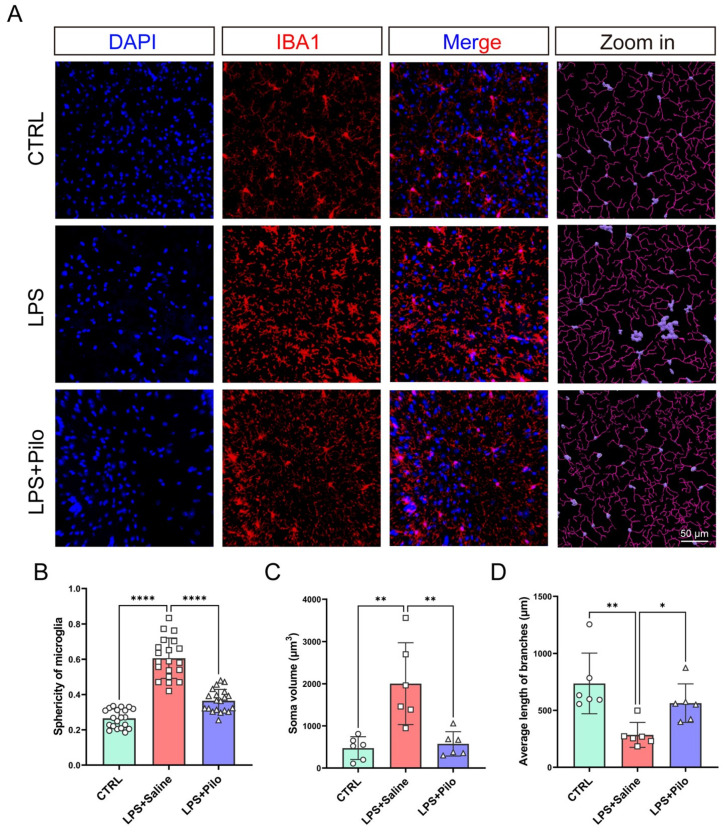
The morphological changes in microglia were attenuated by the activation of muscarinic receptors. (**A**) The brains from the control and LPS-injected mice treated with saline or pilocarpine were stained with DAPI (blue) and anti-Iba1 antibodies (red). Scale bar = 50 μm. The morphology of microglia was evaluated through quantification of cell sphericity (*n* = 20 cells) (**B**), soma volume (**C**), and branch length (*n* = 6 slices) (**D**). * *p* < 0.05, ** *p* < 0.01, **** *p* < 0.0001. Pilo, pilocarpine.

In addition to the changes in cell complexity, morphological characteristics were also altered in activated microglia, mainly including cell sphericity, soma volume, and mean branch length. In order to better measure the microglial morphological changes, we performed 3D imaging on the brain slices of each group of mice. The 3D rendering shown in Figure 7A represented a remarkable disparity in the soma volume (displayed in lavender) between the LPS-induced mice and the other two groups of mice.

Then, we analyzed the above three indicators of microglia in the brains from the control and LPS-stimulated mice treated with saline or pilocarpine. The statistical results showed that the sphericity of microglia increased from 0.27 ± 0.05 to 0.61 ± 0.11 (Figure 7B), and the soma volume was more than 4 times larger due to neuroinflammation (Figure 7C). Under the influence of pilocarpine, these two morphological characteristics recovered to 0.37 ± 0.06 in cell sphericity and 574.2 ± 289.3 in soma volume. Meanwhile, the average length of branches decreased from 737.2 ± 266.4 to 284.7 ± 109.5 after LPS stimulation, which was consistent with the Sholl curves of each group of microglia (Figure 7D) and returned to 563.5 ± 169.4 after the activation of mAChRs. With improvement in the three aforementioned morphological characteristics, the administration of pilocarpine attenuated the microglial morphology in an inflammatory state.

Collectively, the variation in microglial morphology depicted a dynamic change in cell retraction and extension and proved the anti-inflammatory effect of the activation of muscarinic receptors in microglia from another aspect.

### 2.6. Activation of Muscarinic Receptors Protected against Neuronal Damage through Direct and Indirect Effects

Neuroinflammation often leads to fatal damage to neurons, causing neurological deficits such as dementia, stroke [35], and motor dysfunction [36]. In response to external infections or chronic inflammatory stimuli, the sustained activation of glial cells results in an excessive production of inflammatory cytokines, which further induce a decline in neuronal activity or even cell death. According to the relevant literature, activating neuronal muscarinic receptors has a protective effect on neurons themselves [37]. In this study, we discovered that stimulating central microglial mAChRs inhibits the secretion of their inflammatory cytokines. Therefore, we believe that the activation of mAChRs may protect neurons through multiple pathways.

**Figure 8:**
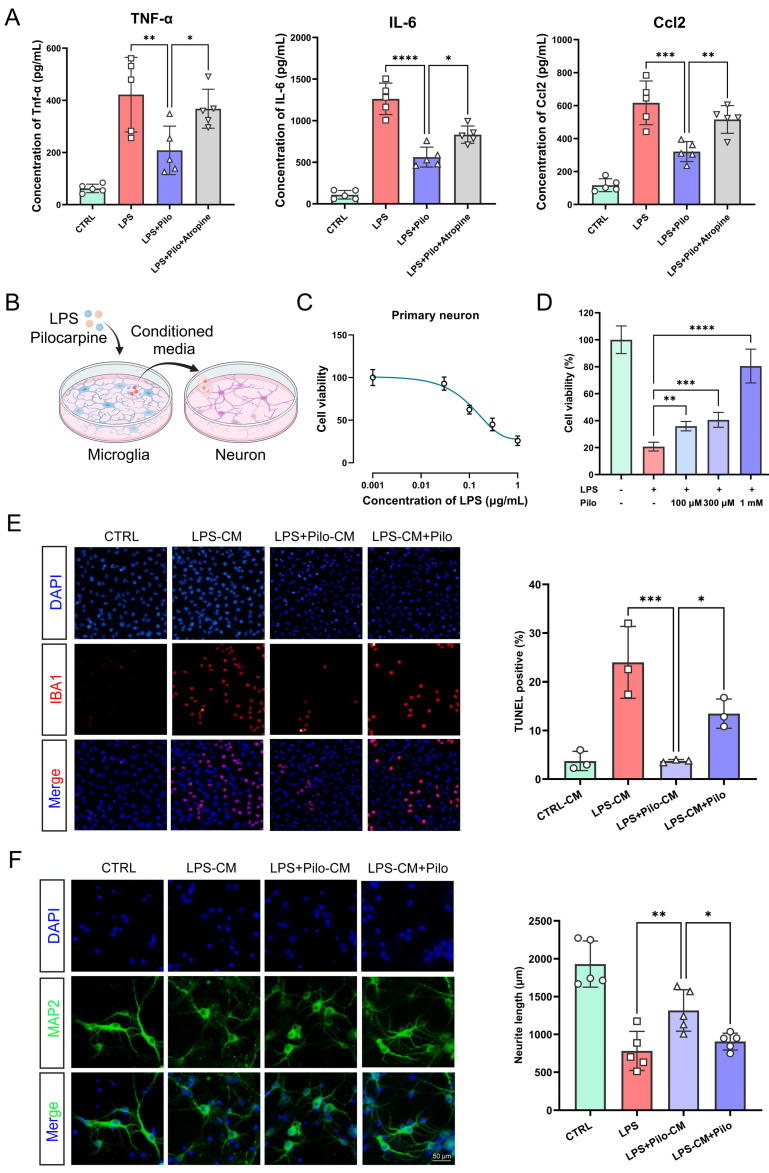
The activation of muscarinic receptors exerted neuroprotective effects through microglia-dependent and -independent ways. (**A**) The expressions of the pro-inflammatory cytokines TNF-α, IL-6, and Ccl2 in primary microglia were examined by the ELISA assay. (**B**) The experimental design for examining the impact of microglia-derived cell supernatant on neuronal vitality. (**C**,**D**) The influence of LPS and pilocarpine on the cell viability of primary neurons was detected by the CCK-8 assay. (**E**) Primary neurons exposed to microglia-conditioned media were stained with DAPI (blue) and TUNEL (red) and displayed the protective effect of the activation of muscarinic receptors on neurotoxicity. Scale bar = 50 μm. (**F**) Primary neurons exposed to microglia-conditioned media were stained with DAPI (blue) and MAP2 (green), and the neurite length was analyzed. Scale bar = 50 μm. All data represent mean ± SD from three or five different experiments. * *p* < 0.05, ** *p* < 0.01, *** *p* < 0.001, **** *p* < 0.0001. Pilo, pilocarpine.

To validate the potential neuroprotective effects of mAChRs on microglia, we first detected the expression levels of neurotoxic inflammatory factors produced by activated microglia. The ELISA results revealed that the expression levels of TNF-α, IL-6, and Ccl2 were downregulated to varying degrees after activation of mAChRs, indicating that these receptors exerted anti-inflammatory effects in microglia (Figure 8A). Remarkably, with Atropine, an antagonist of mAChRs, the inhibition of these cytokines reversed, further substantiating the crucial role of mAChRs in the inflammatory response of microglia.

Subsequently, we examined the impact of microglia-derived cell supernatant on neuronal vitality (Figure 8B). The experimental results revealed the neurotoxicity of the cell supernatant obtained from LPS-stimulated microglia positively correlated with LPS concentration (Figure 8C), a phenomenon potentially associated with elevated levels of secreted inflammatory cytokines. Based on the CCK8 assay, we fitted inhibition curves and selected 1 μg/mL LPS as an inducement concentration for treatment with varying concentrations of pilocarpine. The results indicated a significant reduction in the toxicity of the microglia supernatant to neurons after pilocarpine treatment; neuronal cell vitality recovered from 20.69 ± 3.27% to 80.46 ± 12.51% at a concentration of 1 mM (Figure 8D). These findings suggest that the activation of microglial mAChRs reduced neuronal toxic damage by downregulating the levels of inflammatory cytokines.

To further elucidate the neuroprotective role of microglial mAChR activation in neuroinflammation, we compared the effects of microglia supernatants treated with both LPS and pilocarpine, versus a mixture of LPS-stimulated microglia supernatant and pilocarpine, on neuronal culture. Observations of neuronal cell damage and changes in neurite length were conducted. The TUNEL assay demonstrated that the activation of mAChRs, mediated by microglia as well as directly on neurons, significantly reduced neuronal apoptosis (Figure 8E). Additionally, immunofluorescence labeling of neuronal morphology showed that conditioned media from LPS-stimulated microglia significantly affected neurite length, with decreased MAP2 fluorescence intensity and average neurite length. Conversely, the activation of mAChRs on microglia and neurons enhanced neurite lengths from 780.9 ± 258.1 to 1317.0 ± 274.3 for microglia and from 780.9 ± 115.4 to 1252 ± 189.7 for neurons (Figure 8F). Notably, compared to direct activation of mAChRs on neurons, activation on microglia displayed more remarkable reverse on neural damage, suggesting that beyond direct protective effects on neurons, mAChR activation can improve the inflammatory environment through microglia, thereby reducing the central concentration of neurotoxic substances and reversing neuronal death.

## 3. Discussion

Neuroinflammation, a complicated neurological immune response primarily mediated by activated microglia, is increasingly recognized as a significant factor in the etiology and pathology of neurodegenerative and cerebrovascular diseases [35]. In the early stages of disease, as the primary immune defense of the CNS, the activation and adaptive functional changes in microglia enable them to eliminate pathogens by releasing inflammatory factors and toxic substances. However, under persistent inflammatory conditions, due to the secretion of a large number of inflammatory factors, pathological proliferation, and excessive phagocytic function, overactivated microglia demonstrate a significant neurotoxicity with excessive synaptic pruning [38]. Therefore, intervention in the pathological phenotype of microglia under inflammatory conditions has a positive effect on many neurological diseases.

Studies have shown that agents targeting the muscarinic receptors, such as agonists or cholinesterase inhibitors, exhibit significant therapeutic effects on neurodegenerative diseases. Despite the previous literature suggesting that agonism of mAChRs directly enhances neuronal activity [39], our study revealed a significant reduction in central inflammation levels post-administration, indicating that pilocarpine has a regulatory effect on CNS cells other than neurons, particularly microglia that secrete inflammatory factors. This indicates that pilocarpine also regulates other cells in the CNS, especially microglia with inflammatory factor secretion functions [40]. Combined with in vitro validation experiments, we report here that pilocarpine exerts anti-inflammatory effects in microglia, offering a new approach to therapeutic strategies for neuroinflammation and other related neurological diseases.

To elucidate the impact of activating mAChRs on microglial cells within the central cytokine milieu, we examined a range of cytokines from different cellular origins. This included a series of microglia-derived cytokines with anti-inflammatory and pro-inflammatory functions [41], chemokines that recruit immune inflammatory infiltrates [42], and non-microglial cell-derived cytokines that participate in tissue homeostasis [43]. GM-CSF, which is mainly produced by lymphocytes, has a profound role in immunoregulation through the modulation of microglial phenotypes [44]. In experimental autoimmune encephalomyelitis (EAE), T cell-derived GM-CSF leads to the activation and M1 polarization of microglia and exacerbates CNS inflammatory responses [45]. Another lymphocyte-derived cytokine, IFN-γ, also directly acts on microglia to amplify the neurotoxic effects in inflammatory states through inducing oxidative stress [46]. The results demonstrated that the expression levels of GMCSF and IFN-γ remained unchanged in the brains and spinal cords, indicating that the primary changes following administration were related to the inflammatory response of microglial cells. Although factors secreted by other immune cells have been shown to regulate the inflammatory phenotype of microglia [47], their expression levels in the early stages of the CNS inflammation have not yet changed. At the initial stage of neuroinflammation, the T-cell-dominated adaptive immunity does not have a significant impact on the CNS.

Then, we conducted additional in vitro experiments to verify that both the central commonality of these detected factors and the activation of the NF-κB signaling pathway are regulated by the activation of mAChRs in primary microglia. When central inflammation develops to a certain extent, it damages the BBB [48], leading to an increase in peripheral immune inflammatory infiltration and thereby promoting the deterioration of neurological diseases. In this study, we found that during the early stages of neuroinflammation, central inflammation is dominated by the inflammatory response of microglia. The mAChRs on microglia, which play an anti-inflammatory role, can be activated by the neurotransmitter acetylcholine. This mechanism indicates that the crosstalk between microglia and neurons is an important target for early neuroinflammation treatment. In addition to the inhibitory effects on pro-inflammatory cytokines and signaling pathways, we observed that mAChRs have a regulatory role on the expression levels of the anti-inflammatory cytokine IL-10, which has a crucial role in limiting the immune response to pathogens, as well as preventing damage to the host [49]. It is reported that the microglia-derived IL-10 accelerates post-intracerebral hemorrhage hematoma clearance [50], reduces ROS production, and prevents neuroinflammation and neurodegeneration [51]. More than that, astrocytes [52], one of the types of CNS-resident cells, also secrete IL-10 in the progression of neuroinflammation and alter microglial polarization to the M2 phenotype, which is protective and immunosuppressive. According to our current research, the expression of IL-10 in both the brain and spinal cord tissues of the neuroinflammation mouse model and LPS-stimulated primary microglia was significantly decreased following the activation of mAChRs. Despite being another major source of IL-10 in the CNS, whether the immunological response of astrocytes changes after pilocarpine administration and whether their levels of IL-10 expression also undergo downregulation require further detection and analysis.

Subsequently, we analyzed the distribution density and morphological characteristics of microglia, including the average fluorescence intensity of Iba1, the number of cell branches, and the sphericity and volume of the cell body. Our findings suggest that the activation of mAChRs significantly influences the phenotypes of microglia during CNS inflammation. Furthermore, this study confirmed the anti-inflammatory effect of mAChRs by observing reduced expression levels of pro-inflammatory factors and increased levels of anti-inflammatory factors following drug administration.

In neuroinflammation, the causes of neuronal damage are complex and diverse. These include disturbed transport balance due to higher ATP demand [53], the accumulation of the presynaptic protein bassoon [54], deficits in the transport of synaptic cargoes [55], pathological-calcium-channel-activity-induced calcium accumulation, arrest of mitochondrial transport, and alteration of the frequency of neurotransmitter release from presynaptic terminals [56]. Dysfunction of microglia is also a significant factor. Hyperactivated microglia exert influence on neuronal activity and physiological functions through the pathological phenotypes of secreting neurotoxic inflammatory factors, tissue gliosis, and abnormal shearing of synapses. Therefore, alleviating microglial pathology is one of the targets for neuroprotective therapy.

In previous studies, researchers often attributed the neuroprotective effects of mAChR agonists to their direct stimulation of neurons. However, in this study, we further propose their regulation on microglial phenotypes and indirect protective effects on neurons. To verify the protective effect of microglia-mediated muscarinic receptor activation on neurons, we treated neurons with conditioned medium from microglia stimulated by LPS. The detection results of relevant indicators reflecting the physiological state of neurons, including neuronal viability, apoptosis levels, and neurite length, demonstrated that the supernatant of microglia treated with both the mAChR agonists—pilocarpine and LPS—significantly reduced the impact on neuronal activity compared to the LPS group alone, with a noticeable decrease in neurotoxicity. A further comparison with the direct stimulation of mAChRs was conducted by collecting the supernatant after LPS stimulation and adding additional pilocarpine to achieve a final concentration of 1 mM, which was then applied to neurons. The results demonstrated that, in comparison to its direct effect on neurons, the intervention of pilocarpine on the inflammatory phenotype of microglia exhibits greater benefits. Upon comparing the degrees of neuronal apoptosis and the length of neurites under two different treatments, the differences were found to be statistically significant. This analysis indicated that the activation of mAChRs mediated by microglia played a significant role in their neuroprotective functions, yet this role has been overlooked.

Based on the alleviation of microglial inflammatory phenotypes following pilocarpine administration, mAChRs not only contribute to repair after neurological damage but also serve as crucial targets for mitigating central inflammation and preventing neural injuries. These findings provide a theoretical basis for expanding the clinical indications of mAChR agonists. Additionally, the specific downstream mechanisms of mAChRs may offer clues for the development of inflammation-related targets, warranting further research.

## 4. Materials and Methods

### 4.1. Animals and LPS-Induced Neuroinflammation Model

C57BL/6J mice, 8–10 weeks old (25–30 g), were purchased from Shanghai Laboratory Animal Center. The mice were housed in a 12 h light/dark cycle at a constant temperature and humidity-controlled room for a minimum of 3 days before surgery, with free access to food and water. In the present study, all the mice were randomly assigned to the following experiments. The experimental design is shown in wester 1. An LPS-induced CNS inflammation model was described previously [57]. In the current study, 10-week-old mice were injected intraperitoneally (i.p.) with LPS at a dose of 2 mg/kg. All animal experiments in this study were approved by the Animal Care and Use Committee of Shanghai University of Traditional Chinese Medicine (PZSHUTCM2311120002).

### 4.2. Cell Culture and Treatment

With mild modifications, primary mouse hippocampal neurons were prepared as previously described. Neurons were dissected from C57BL/6J mice at postnatal day 1 (Sprague-Dawley, Slac Laboratories, Chinese Academy of Sciences). The embryos were collected, and the hippocampi were dissected in ice-cold Hank’s buffered salt solution without Ca^2+^ and Mg^2+^ (GIBCO, Grand Island, NY, USA). Dissected pieces of hippocampus tissue were pooled together and transferred to an enzymatic dissociation medium containing 0.05% trypsin-EDTA (Gibco) and then incubated for 6–8 min at 37 °C. After enzymatic dissociation, the trypsin solution was aspirated, and the tissue was triturated with a sterile Pasteur pipette in complete Minimum Essential Medium (DMEM, 10% FBS (*v*/*v*), 2 mM L-glutamine, 1% Penicillin-Streptomycin (*v*/*v*) (GIBCO, 15070063)) plus 2000 IU/mL DNase. After 800 g of the mixture underwent 8 min of centrifugation, the pellets were resuspended in complete DMEM/F12 medium and plated on poly-L-lysine pre-coated 96-well or 24-well plates. The cells were cultured in a humidified atmosphere (5% CO_2_–95% room air) at 37 °C for 2 h; then, the medium was replaced with neurobasal medium plus 2% B27 and 2 mM Gluta-MAX supplements. The purity of the neurons was around 90%, as indicated by Hoechst staining for nuclei and β-tubulin staining for neural cell body and neurites.

Primary mouse microglia were isolated from postnatal day 1 C57BL/6J mice (Slaccas, Shanghai, China) and cultured in F-12 (Gibco, 11765-054) with 10% heat-inactivated FBS and 1% Penicillin/Streptomycin, at 37 °C in a humidified incubator under 5% CO_2_. Human embryonic kidney 293T (HEK293T) cells, obtained from ATCC, were cultured in DMEM with 10% FBS, 1% L-Ala-Gln, and 1% penicillin/streptomycin at 37 °C in a humidified incubator under 5% CO_2_.

Lipopolysaccharides (LPSs) (Sigma-Aldrich, Darmstadt, Germany, L4391) and pilocarpine (MedChemExpress, Shanghai, China, HY-B0726) were dissolved in PBS. The concentration of LPS used for mouse primary microglia was 1 μg/mL.

### 4.3. Enzyme-Linked Immunosorbent Assay (ELISA)

Primary microglia were plated into 24-well plates and then treated with 1 mM pilocarpine, along with 1 μg/mL LPS stimulation for 24 h. Cell supernatants were collected and centrifuged to remove cell debris sediment.

Lysates were prepared from frozen tissues homogenized in RIPA buffer, with protease/phosphatase inhibitors and DNase I (1:500 dilution; Sangon, Shanghai, China, A510099). The expression of pro-inflammatory cytokines and chemokines were analyzed using mouse TNF-α (Multisciences, Hangzhou, China, EK282/4-96), mouse IL-6 (Multisciences, EK206/3-96), and mouse Ccl2 (Multisciences, EK106/2-96) ELISA kits according to the manufacturer’s instructions. The results of the ELISA assay were normalized to the protein concentration examined by the BCA assay.

### 4.4. RNA Extraction and Quantitative Reverse-Transcription Polymerase Chain Reaction (RT-qPCR) Analysis

Total RNA was extracted from primary microglia using the TaKaRa MiniBEST Universal RNA Extraction Kit (Takara, Kyoto, Japan, 9767) and quantified using NanoDrop OneC (Thermo Fisher Scientific, Waltham, MA, USA). cDNA was generated using the PrimeScript™ RT reagent Kit with gDNA Eraser (Takara, RR047A). Quantitative real-time PCR (qPCR) was performed for each sample in triplicate on the StepOnePlusTM Real-Time PCR System (Applied Biosystems, Waltham, MA, USA, 4376600) using the TB Green^®^ Rremix DimerEraserTM (Takara, RR091A). The primers for real-time PCR amplification were designed with Primer Premier 6. The primer pair sequences were matched by BLASTn to the genome sequence to identify the primer locations with respect to the exons. A comparative threshold cycle (CT) method was used to analyze the real-time PCR data, where the amount of target, normalized to an endogenous reference of 18 s (∆CT) and relative to the calibrator of untreated control (∆∆CT), was calculated by equation 2^−∆∆CT^. The sequences of the PCR primers were shown in Table 1:

### 4.5. Tissue Preparation for Cryotomy

The mice were deeply anesthetized with sodium pentobarbital via intraperitoneal injection at a dose of 60 mg/kg. Afterwards, the mice were perfused with cold PBS for 5 min, followed by 4% paraformaldehyde solution for 15 min. Then, the brains and spinal cords were excised and postfixed overnight in 4% paraformaldehyde at 4 °C and dehydrated in 30% sucrose–PBS. The brains and spinal cords were mounted in optimal cutting temperature embedding medium, frozen, and cut coronally at a 30 μm thickness on a cryostat.

### 4.6. Morphological Analysis

A Sholl analysis was manually performed for each cell by drawing concentric circles using ImageJ v1.54f, centered on the soma, increasing 1 μm with every circle [58], and then counting the number of intersections between microglia branches and each increasing circle to create a Sholl plot. The area under the curve (AUC) was further calculated to numerically compare cell complexity between different groups.

### 4.7. Cell Viability Assay

Primary microglia and neuron cultures were maintained for 6 days in culture. Primary microglia were activated by LPS at different concentrations (0.03, 0.1, 0.3, and 1 μg/mL). Based on the pilot dose–effect study, subsequent experiments were carried out by pretreatment with pilocarpine at different concentrations (0.1, 0.3, and 1 mM), followed by 1 μg/mL LPS for 24 h. Cell viability was evaluated using a rapid colorimetric assay. In brief, the primary hippocampal neurons were seeded at 10^5^ cells per well on a 96-well microplate. After 6 days, the cells were treated with the conditioned medium of microglia in the presence of pilocarpine. After 24 h of treatment, CCK-8 solution was added into each well; this was followed by incubation for indicated periods. Cell viability was determined by measuring the OD at 450 nm.

### 4.8. Western Blot

Tissue extracts of brains and spinal cords were collected in lysis buffer [1% 100 mM Phenylmethanesulfonyl fluoride (Beyotime, Suzhou, China, ST506), 1% Protease Inhibitor (Apexbio, Houston, TX, USA, K1007), 1% Phosphatase Inhibitor Cocktail (Apexbio, K1015-A), and 1% Phosphatase Inhibitor Cocktail (Apexbio, K1015-B), supplemented with RIPA (Epizyme, Shanghai, China, PC101)] and denatured by heating at 95 °C for 10 min. A total of 20 μg of proteins were subjected to BeyoGel™ Plus PAGE precast gel (Beyotime, P0451S) and transferred onto Immobilon^®^-P PVDF membranes (Sigma-Aldrich, IPVH00010). The membranes were blocked for 1 h at room temperature in protein-free rapid blocking buffer (Epizyme, PS108P) and immunoblotted with anti-NF-κB/p65 (CST, Danvers, MA, USA, 8242S), anti–-phospho–NF-κB p65(Ser536) (CST, 3033T), and anti-Actin (Sangon, C650044). The membranes were incubated with the indicated primary antibodies overnight at 4 °C and then incubated with goat anti-rabbit (Biotime, Suzhou, China, A0208) or anti-mouse immune globulin G–horseradish peroxidase (Proteintech, Wuhan, China, SA00001-1) secondary antibodies for 2 h at room temperature. The proteins were eventually visualized by Amersham Image Quant 800 (Cytiva, North Logan, UT, USA, 29399481); the band intensity of Western blots was quantified with the open-source ImageJ.

### 4.9. Immunofluorescence Staining

Primary microglia were fixed with 4% paraformaldehyde (PFA) for 15 min at room temperature and permeabilized with 0.5% Triton (Sigma-Aldrich, 9036-19-5) for 45 min at room temperature. After washing three times with PBS, the cells were blocked by 5% BSA (Solarbio, Beijing, China, A8020) in PBS for 30 min. Next, the cells were incubated with anti-Iba1 (Wako, Tokyo, Japan, 019-19741) for 2 h at room temperature, washed three times with PBS, and incubated with Alexa Fluor secondary antibodies (Abcam, Cambridge, UK, ab150077) for 2 h at room temperature in the dark. Washed three times with PBS, the cells were stained with 4,6-diamidino-2-phenylindole (DAPI) (Sangon, E507303) for 15 min in the dark and then washed three times with PBS. Confocal images were obtained with a confocal microscope system (Nikon, Tokyo, Japan, A1RMP+).

### 4.10. TUNEL Staining

For quantification of neuronal apoptosis by inflammation, a terminal deoxynucleotidyl transferase dUTP nick end labeling (TUNEL) staining assay was performed using a One-Step TUNEL Apoptosis Assay Kit (Beyotime, C1089). Primary neurons were plated into 24-well plates and cultured on a poly-lysine-coated slide and then divided into four groups with the treatment of the conditional medium of microglia. After 24 h of incubation, the cells were fixed with 4% paraformaldehyde, permeabilized with 0.3% Triton X-100, and stained with Cy3-labeled dUTP according to the manufacturer’s instructions. Washed two times with PBS, the cells were stained with DAPI for 15 min. Coverslips were then mounted, and the samples were observed under a laser scanning confocal microscope (Leica SP8; Leica, Wetzlar, Germany). The data were expressed as the ratio of TUNEL positive neurons normalized by the fluorescence intensity of DAPI (%).

### 4.11. Statistical Analysis

All data were expressed as mean ± standard deviation (SD). The data were normally distributed, as tested using the Shapiro–Wilk test (*p* > 0.1). For comparisons between two groups, Student’s *t* test was used for comparisons of variables with normal distribution from independent samples. Multiple comparisons were statistically analyzed using a one-way analysis of variance (ANOVA) followed by Dunnett’s test. The data analyses were conducted using Prism 9 (GraphPad Software, San Diego, CA, USA). All statistical tests were two-sided, and *p* < 0.05 was considered statistically significant. All the experiments were performed three or more times.

## 5. Conclusions

In summary, this study revealed that the activation of mAChRs modulated the expression levels of various inflammatory and chemotactic factors in the brain and spinal cord of a neuroinflammation mouse model. In addition to the direct excitatory protection in neurons, the modulation of the phenotypes of microglia, including morphology, distribution, and levels of inflammation, plays a primary role. Such dual neuroprotective mechanisms underscore the significance and diversity of central mAChR functions, suggesting potential applications in inflammation-related neurological disorders.

## Figures and Tables

**Table 1:** List of RT-qPCR primer sequence details.

Gene Name ^1^	Primer Sequence (5′–3′)
IL-1β	Forward: GAGACTTCCATCCAGTTGCCTTCT Reverse: GTGTAATTAAGCCTCCGACTTGTGAAG
IL-4	Forward: GAGACTTCCATCCAGTTGCCTTCT Reverse: GTGTAATTAAGCCTCCGACTTGTGAAG
IL-6	Forward: GAGACTTCCATCCAGTTGCCTTCT Reverse: GTGTAATTAAGCCTCCGACTTGTGAAG
IL-10	Forward: GAGACTTCCATCCAGTTGCCTTCT Reverse: GTGTAATTAAGCCTCCGACTTGTGAAG
IL-17	Forward: GAGACTTCCATCCAGTTGCCTTCT Reverse: GTGTAATTAAGCCTCCGACTTGTGAAG
IL-23	Forward: GAGACTTCCATCCAGTTGCCTTCT Reverse: GTGTAATTAAGCCTCCGACTTGTGAAG
TNF-α	Forward: GGAACTGGCAGAAGAGGCACTC Reverse: GGAATGAGAAGAG-GCTGAGACATAGG
GM-CSF	Forward: GCUUAUCAUCAGAAAGGUA Reverse: UACCUUUCUGAUGAUAAGC
IFN-γ	Forward: GCAUCAUCGUUUCCUACAA Reverse: UUGUAGGAAACGAUGAUGC
Ccl2	Forward: GCAUCAUCGUUUCCUACAA Reverse: UUGUAGGAAACGAUGAUGC
Ccl7	Forward: GAGACTTCCATCCAGTTGCCTTCT Reverse: GTGTAATTAAGCCTCCGACTTGTGAAG

^1^ Abbreviations: IL, interleukin; Tnf, tumor necrosis factor; GM-CSF, granulocyte–macrophage colony-stimulating factor; IFN, interferon.

## Data Availability

The data presented in this study are available on request from the corresponding authors.

## Supplementary Materials

The following supporting information can be downloaded at https://www.mdpi.com/article/10.3390/ijms251910432/s1.
