# Supplementary material for: The Activation of Muscarinic Acetylcholine Receptors Protects against Neuroinflammation in a Mouse Model through Attenuating Microglial Inflammation
Source: Int J Mol Sci. 2024 Sep 27;25(19):10432. doi: 10.3390/ijms251910432 (PMC11476571; doi:10.3390/ijms251910432)

Figure S1. The western blotting results from three repeated experiments of the activation of NF-κB pathway in the brains of mouse models.

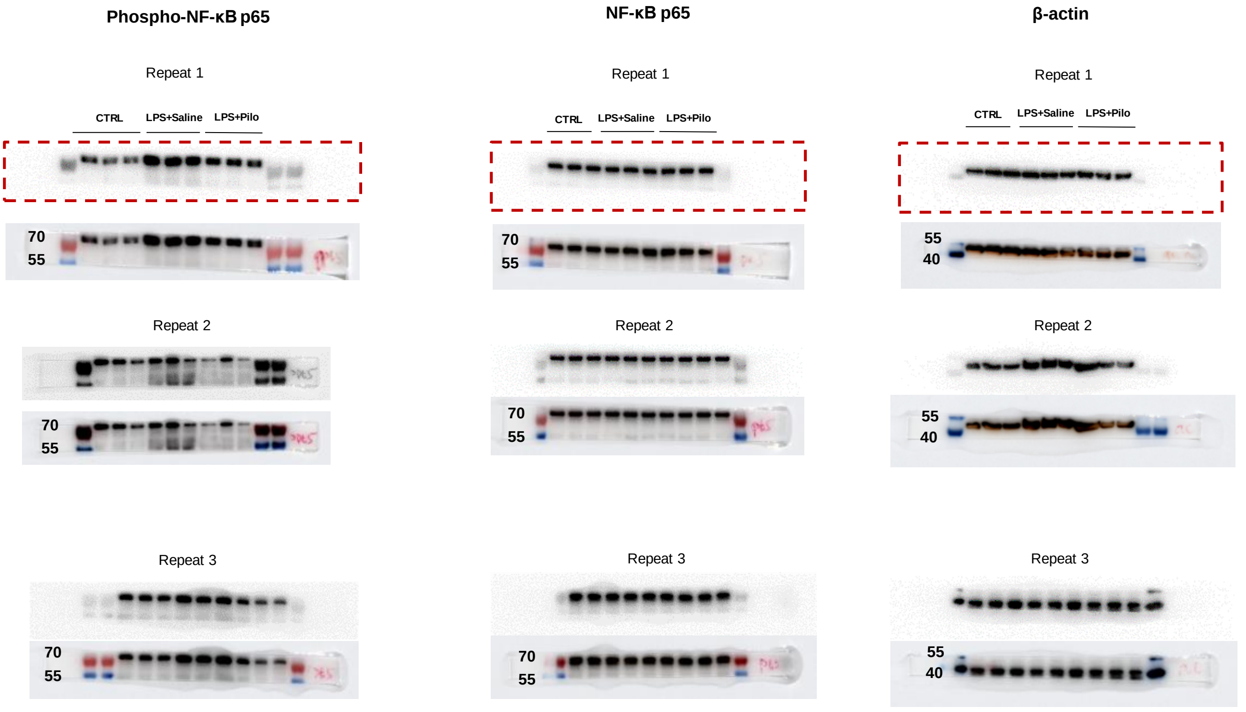

Figure S2. The western blotting results from three repeated experiments of the activation of NF-κB pathway in the spinal cords of mouse models.

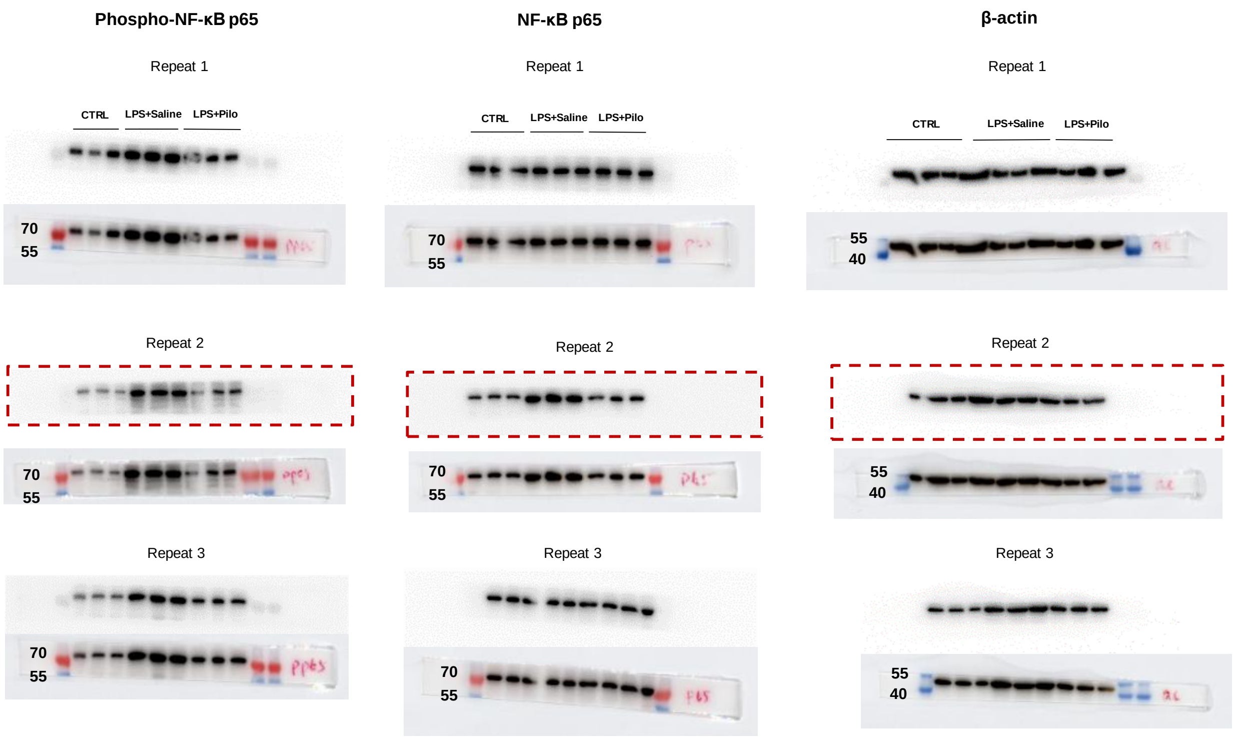

Figure S3. The western blotting results from three repeated experiments of the activation of NF-κB pathway in the primary microglia.

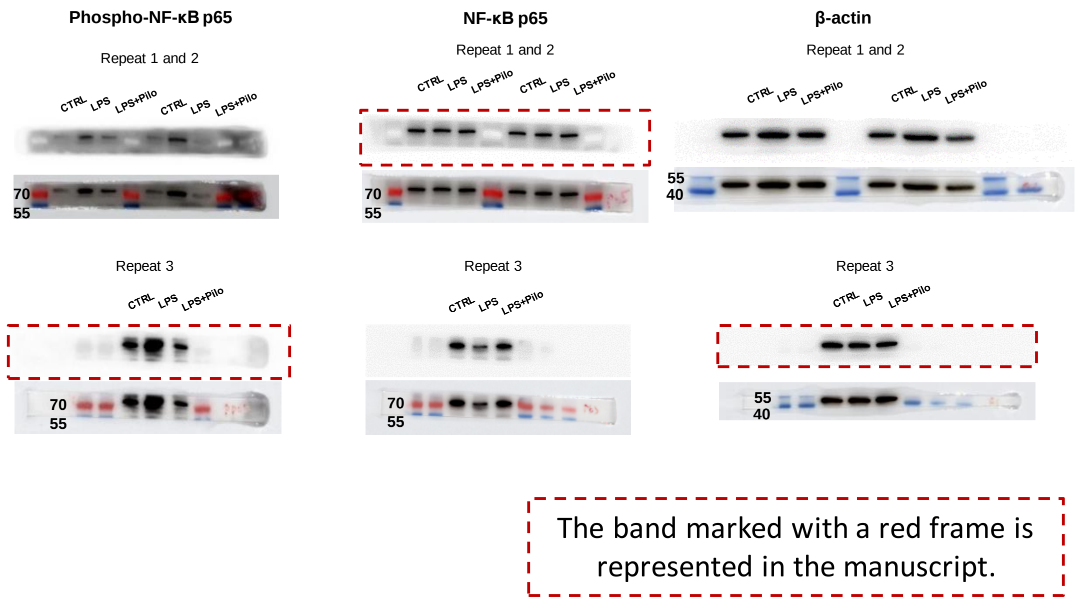

Supplement: Supplementary file 1 [file ijms-25-10432-s001.zip › ijms-3209661-supplementary.pdf]
